# Atrial thrombus as a complication of SLE and APS in an 8-year-old child

**DOI:** 10.1186/s12969-020-00484-z

**Published:** 2020-11-17

**Authors:** Hai-bo Yan, Yu-mei Li

**Affiliations:** Department of Pediatric Critical Medicine, The First Hospital of Jilin University, 71 Xin Min Street, Changchun, 130000 China

**Keywords:** Systemic lupus erythematosus, Nephrotic syndrome, Anticardiolipin antibody syndrome, Atrial thrombus

## Abstract

**Background:**

Systemic lupus erythematosus (SLE) is an autoimmune disease involving multiple systems with various clinical manifestations. Renal involvement is common, but intracardiac thrombus is rarely reported as a complication of antiphospholipid syndrome (APS, also known as anticardiolipin syndrome). Anticoagulant therapy is the first-line treatment, and surgery is performed in severe cases. We report a case to improve clinicians’ understanding of disease diagnosis.

**Case presentation:**

An 8-year-old girl was admitted to our hospital because of left costal pain, hematuria and fever. She had obvious edema occult blood 3+, urinary protein 3.2 g/24 h, albumin 17.6 g/L, and total cholesterol 7.21 mmol/L, consistent with a diagnosis of nephrotic syndrome. We continued to track the etiology of nephrotic syndrome and performed a renal biopsy, showing dsDNA 1:10 positivity, low C3, low platelets and hemoglobin, anticardiolipin IgM 12 U/ml, anti-β2-glycoprotein I (β2GPI) 223 U/ml; renal pathology suggested lupus nephritis (LN), and the patient was ultimately diagnosed with SLE, secondary APS and LN. The patient was treated with hormones and immunosuppressants. Sixteen weeks later, her urinary protein was 1+, and the quantity of urine protein was less than 0.5 g/d. Echocardiography showed that the mass in the right atrium was thrombotic. Heparin anticoagulant therapy was effective.

**Conclusion:**

SLE can involve multiple systems and various complications. Thrombus in the right atrium is a rare complication of APS. Early diagnosis and treatment are key to improving the prognosis of children.

## Background

Systemic lupus erythematosus (SLE) is a multisystem autoimmune disease with various clinical manifestations. Lupus nephritis (LN) is a common manifestation of SLE. Anticardiolipin antibody syndrome is easily complicated by thrombi, but intracardiac thrombosis is rare. Here, we report a child with nephrotic syndrome who was then diagnosed with SLE and secondary anticardiolipin antibody syndrome complicated by right atrial thrombus.

## Case presentation

**Fig. 1:**
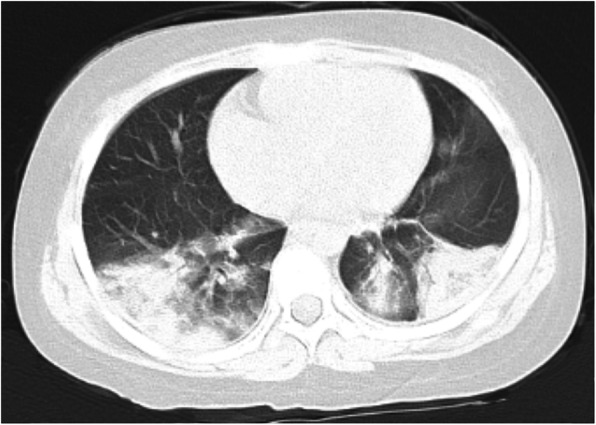
High-density shadows in all lobes of the lungs

**Fig. 2:**
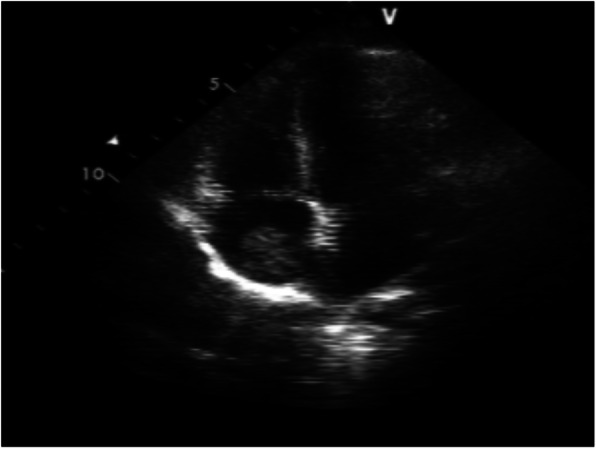
Echocardiography:right atrial thrombus

**Fig. 3:**
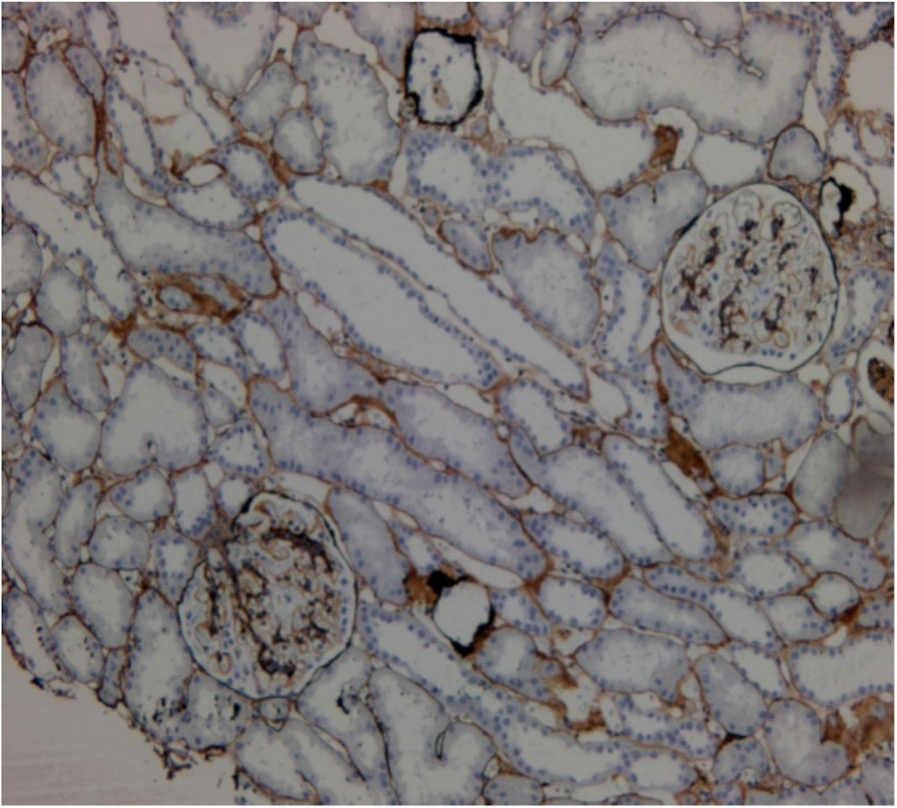
Slight proliferation of mesangial cells and mesangial matrix

**Fig. 4:**
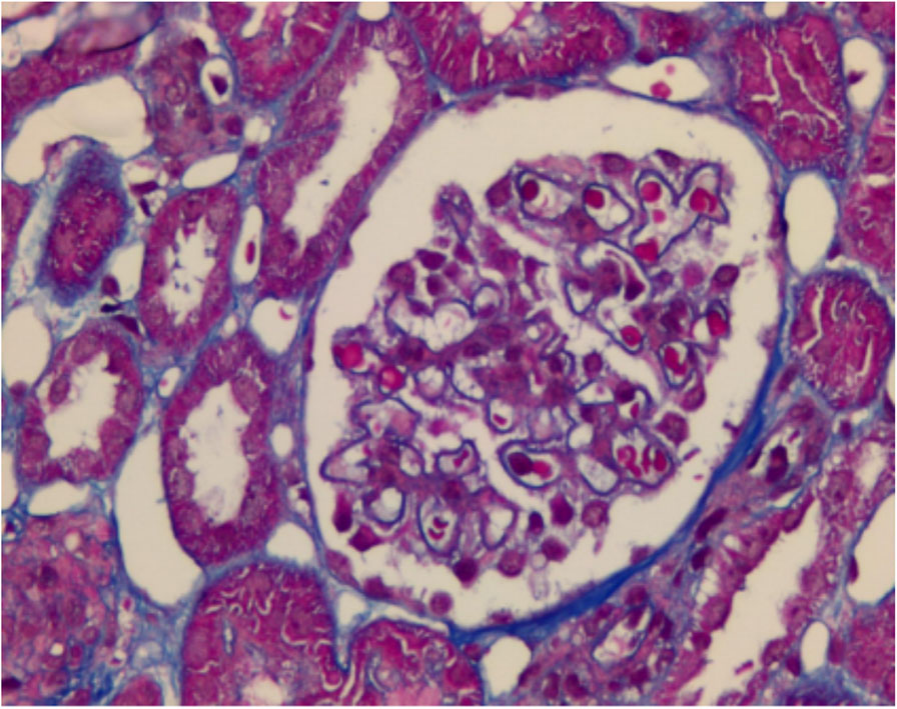
Large amount of furophilic protein deposition subepithelial cell

An 8-year-old female who presented with left rib pain, hematuria and fever was admitted to our hospital. The maximum temperature reached 39.9 °C, with no chills or rash. She was administered cefepime and azithromycin for 1 day, cefotaxime for 5 days, and methylprednisolone for 1 day. On physical examination, her general condition was poor, with eyelid and lower limb edema; coarse respiratory sounds and auscultation of blisters of both lungs were noted, and no other abnormal findings were present at that time. Laboratory findings were as follows: white blood cell count 6.32*10^9^/L, neutrophil 0.48, lymphocyte 0.42, red blood cell count 3.40*10^12^/L, hemoglobin 93 g/L, and platelet count 48*10^12^/L. Her urine revealed 363.69 red blood cells/high-powered field (hpf), protein 3+, 5.1 white blood cells/hpf, erythrocyte sedimentation rate (ESR) 135 mm/h, C-reactive protein (CRP) 25.2 mg/L, albumin 17.6 g/L, total cholesterol 7.21 mmol/L, and urinary protein 3.2 g/24 h. Other laboratory findings included activated partial thromboplastin time (APTT) 138.7 s, direct antiglobulin test positive, ferritin 358.4 μg/L, D-dimer 4937.00 μg/L, and FDG 33.8 μg/ml. Serum showed C3 0.92 g/L (0.9–1.8), C4 0.17 g/L (0.1–0.4), ANA:anti-SSA-60 ±, anti-nRNP/Sm positivity, homogeneous 1:3200 positivity, dsDNA 1:10 positivity, antinucleoome antibody positivity, antimitochondrial M2 positivity, anticardiolipin IgM 12 U/ml (0–10), and anti-β2GPI 223 U/ml (0–20). Bone marrow biopsy revealed secondary anemia, and globular red blood cells accounted for 5.5%. However, respiratory pathogens, myocardial enzymes, mycoplasma pneumoniae/chlamydia antibodies, procalcitonin, folic aci, vitamin B12, reticulocytes, bacterial cultures of blood, stool, tuberculosis spots, Epstein-Barr virus, cytomegalovirus, ANCA were all negative. Hematuria location: urine abnormal red blood cell 60%, urine uniform red blood cell 40%. Bronchoscope results showed inflammation of the endobronchial membrane. Pulmonary CT revealed patchy high-density shadows in all lobes of the lungs, especially in the lower lobes of the lungs (Fig. 1). Color Doppler ultrasonography of the lower limbs ruled out deep vein thrombosis. Abdominal color ultrasound revealed abdominal effusion. Echocardiography showed a kind of round moderate echo with a diameter of approximately 2.0 cm that could be seen at the bottom of the right atrium near the opening of the inferior vena cava. The patient was diagnosed with right atrial thrombus (Fig. 2). Renal pathology using light microscopy revealed 52 glomeruli in the renal tissue; there were 52 glomeruli in the renal tissue, with slight proliferation of mesangial cells and mesangial matrix, swelling and slight proliferation of segmental foot nuclear endothelial cells, slight thickening of glomerular basement membrane and a large amount of phenophilin deposition in subepithelial cells. No microthrombi or crescents were observed, and a small amount of inflammatory cell infiltration dominated by neutrophils was found in some glomeruli. There was slight edema in the renal interstitium, and there was no renal tubular atrophy and obvious inflammatory cell infiltration and fibrosis. No obvious abnormalities were found in the arterioles. Immunofluorescence analysis was positive for IgA, IgM, IgG, C3, C4, and C1q deposited along the glomerular capillary wall and segmental mesangial area. The above features were consistent with membranous LN with mesangial proliferative LN (consistent with types V and II LN) (Figs. 3 and 4). Nephrotic syndrome is a common disease in children that is characterized by edema, massive proteinuria (>50 mg/kg.24 h), hypoproteinemia (<25 g/l) and hypercholesterolemia (>5.7 mmol/l). Our patient’s disease characteristics were consistent with those of nephrotic syndrome [1]. We continued to track the etiology of nephrotic syndrome, and the child had no family history of renal disease; therefore, we investigated secondary factors, including serology results that were positive for ANA and anti-dsDNA with low C3 levels. Although her platelets and hemoglobin levels were significantly lower than normal, she had no rash, arthralgia, photosensitivity, oral ulcer, or butterfly erythema, so a renal biopsy was performed, which suggested LN. The patient was finally diagnosed with SLE and LN. She was treated with pulse methylprednisolone for 3 days and IV diuretic with albumin, followed by oral steroids 1 mg/kg.d after 3 days, with rapid resolution of edema. Anticardiolipin IgM of 12 U/ml and anti-β2GPI of 223 U/ml were consistent with secondary APS. What was the cause of the space-occupying mass in the right atrium? At this point, as no clear etiological evidence was found, a repeated echocardiography indicated that the nature of the right atrial mass was a thrombus. The mass was reduced by anticoagulant therapy with heparin for 2 weeks. By 3 weeks, proteinuria decreased to 1.0 g/day, and by 16 weeks, she was in complete remission with < 0.5 g proteinuria. After 6 months of follow-up, the patient has remained in complete remission, but 2.5 mg of prednisolone is administered each day.

## Discussion

Systemic lupus erythematosus (SLE) is an autoimmune disease that can involve multiple organs. Its pathological manifestations are immune complex deposition and vasculitis changes. Lupus nephritis (LN) is a common manifestation of SLE. In the early stage of SLE, LN may show slight abnormalities in urine (microhematuria, albuminuria) [2–5], with nephrotic syndrome, hypertension, or renal failure [6, 7]. In this case, the child was diagnosed with nephrotic syndrome, and renal biopsy revealed LN. According to the International Society of Nephrology (ISN/RPS), LN is divided into six grades (grades I-VI) [8]. Nephrotic syndrome is usually associated with diffuse or membranous. LN [9]. However, membranous LN or mesangial proliferative LN is rare in children. Echocardiography revealed an occupying mass in the right atrium. The nature of the mass is usually a neoplasm, tumor, or thrombus. The neoplasms are most common on the valves, and no neoplasms were found by echocardiography. Scholars [10] have pointed out that myxoma is the most common cardiac tumor, is usually located in the left atrium, and is usually attached to the atrial septum. Cardiac myxoma is mostly found in the left atrium, which is mostly attached to the atrial septum, accounting for approximately 15% of the right atrium, while atrial thrombus is more common in the left atrial appendage. Thrombi in the right atrium are rare. How can an atrial thrombus and a cardiac myxoma be distinguished? In fact, the identification of the two is mainly determined using ultrasound, and generally speaking, the identification is not very difficult. Cardiac myxoma can be accompanied by blood flow fluctuations, but a thrombus generally is not. The shape of cardiac myxoma can change with the relaxation or contraction of the heart, but a thrombus will not. Anticoagulants can dissolve part of the thrombus, but cardiac myxoma cannot be dissolved by anticoagulants. The volume of the atrial mass was reduced in our patient after anticoagulation with heparin.

Our pediatric patient was diagnosed with APS with a right atrial thrombus; intracardiac thrombosis is a rare complication of antiphospholipid syndrome (APS), and the physiological mechanism is not clear. The hypercoagulable state of nephrotic syndrome can easily lead to thrombosis, and SLE with positive anticoagulant activity or medium or high levels of anticardiolipin antibody can increase the risk of thrombosis or thromboembolism in the heart [11]. Thirty-seven percent of SLE patients with anticardiolipin antibody syndrome were positive for the β2GPI antibody [12], which is an independent risk factor for thrombosis [13]. The chronic inflammatory state of SLE also increases the risk of thrombosis. Some scholars [14] have pointed out that thrombosis is related to the presence of lupus anticogulant (LAC)-positive and anti-RNP/Sm antibodies, and the combined use of LAC and anti-RNP/Sm antibodies as predictors of venous thromboembolism is worth studying. In this case, our patient had a right atrial thrombosis that was associated with secondary APS, which has a higher risk of thrombosis and valvular disease than patients who do not have antiphospholipid antibodies [15]. In addition, others [10, 16, 17] have pointed out that when the thrombus in the cardiac cavity is large and irregular, it easly falls off and leads to a very high risk of recurrent pulmonary embolism. In this patients, pulmonary CT showed inflammation and consolidation changes in both lungs (Fig. 1). The patient was treated with the anticoagulant heparin, and echocardiography showed that the volume of the thrombus was reduced, so no surgery was performed to remove the thrombus. Therefore, for SLE patients with high risk factors for thrombosis, attention should be paid to screen for thrombi and to treat with anticoagulation therapy in a timely manner.

## Conclusions

In summary, we report a rare case of atrial thrombosis caused by secondary APS. Atrial thrombi are rare, and anticoagulant therapy is the first-line treatment. This case report is presented to improve clinicians’ understanding of the disease, to find the real cause behind the disease, early diagnosis and treatment, and to improve the prognosis of patients. For adolescents, when multiple systems are involved, the diagnosis is most likely to be SLE. The diagnosis of SLE can be confirmed by conducting an immunological examination and a renal biopsy as soon as possible.

## Data Availability

Not applicable.

## Abbreviations

SLE: Systemic lupus erythematosus
CRP: C reactive protein
ESR: Erythrocyte sedimentation rate
LN: Lupus nephritis
APTT: Activated partial thromboplastin time
LAC: Lupus anticoagulant
